# Reduced vagal activity in borderline personality disorder is unaffected by intranasal oxytocin administration, but predicted by the interaction between childhood trauma and attachment insecurity

**DOI:** 10.1007/s00702-022-02482-9

**Published:** 2022-03-11

**Authors:** Sarah N. Back, Marius Schmitz, Julian Koenig, Max Zettl, Nikolaus Kleindienst, Sabine C. Herpertz, Katja Bertsch

**Affiliations:** 1 Department of Psychology, Ludwig-Maximilians-University Munich, Leopoldstraße 13, 80802 Munich, Germany; 2 Department of General Psychiatry, Center for Psychosocial Medicine, University of Heidelberg, Heidelberg, Germany; 3 Department of Child and Adolescent Psychiatry, Psychosomatics and Psychotherapy, Faculty of Medicine and University Hospital Cologne, University of Cologne, Cologne, Germany; 4 Institute of Psychosocial Prevention, Center for Psychosocial Medicine, University of Heidelberg, Heidelberg, Germany; 5 Medical Faculty Mannheim, Institute of Psychiatric and Psychosomatic Psychotherapy, Central Institute of Mental Health, Heidelberg University, Mannheim, Germany

**Keywords:** Heart-rate variability, Vagal activity, Borderline personality disorder, Attachment, Oxytocin, Early life adversity, Childhood trauma

## Abstract

Individuals with borderline personality disorder (BPD) show self-regulatory deficits, associated with reduced heart-rate variability (HRV). However, results on reduced HRV in BPD remain heterogeneous, thus encouraging the search for developmental constructs explaining this heterogeneity. The present study first examined predictors of reduced resting-state HRV in BPD, namely the interaction between self-reported adult attachment insecurity and childhood trauma. Second, we investigated if alterations in resting-state HRV are modified by intranasal oxytocin administration, as oxytocin may enhance HRV and is implicated in the interaction between childhood trauma and disturbed attachment for the pathogenesis of BPD. In a randomized, placebo-controlled trial, 53 unmedicated women with BPD and 60 healthy controls (HC) self-administered either 24 I.U. of oxytocin or placebo and underwent a 4-min electrocardiogram. Our results replicate significantly reduced HRV in women with BPD, explained up to 16% by variations in childhood trauma and attachment insecurity. At high levels of acute attachment insecurity, higher levels of childhood trauma significantly predicted reduced HRV in BPD. However, our results do not support a significant effect of oxytocin on mean HRV, and no interaction effect emerged including childhood trauma and attachment insecurity. Our findings highlight a complex interaction between reduced vagal activity and developmental factors in BPD.

## Introduction

Borderline personality disorder (BPD) is a severe mental disorder, which affects around 15–28% patients in clinical populations (APA 2001). Core symptoms of BPD are emotional dysregulation, impulsivity, and interpersonal hypersensitivity, all of which have been shown to be associated with alterations in heart-rate-variability (HRV; Lane et al. 2009; Thayer and Brosschot 2005; Ottaviani et al. 2018; Weise et al. 2020a).

HRV is defined as the beat-to-beat variability of heart rate (Berntson et al. 1997) indicating different autonomic states (i.e. higher variability indicating majorly vagal and lower variability reflecting majorly sympathetic predominance of the autonomic nervous system; ANS). While the sympathetic system is associated with energy mobilization during stress, the parasympathetic system reflects vegetative and restorative functions (Thayer and Brosschot 2005). According to the model of neurovisceral integration, these two states are in dynamic balance and interplay with different environmental demands (Thayer and Brosschot 2005). Chronic autonomic imbalance, in which the sympathetic stress system is hyperactive and the parasympathetic system is hypoactive (e.g., lower HRV at rest), in turn, is theorized to be associated with various pathological conditions (Thayer and Brosschot 2005), including BPD (for meta-analysis, see Koenig et al. 2016). Indeed, unaltered HRV has been proposed as a biomarker of self-regulation and mental health (Beauchaine and Thayer 2015). With regard to the aetiology of BPD, various results on reduced HRV point to a chronic reduction of the vagal ANS component, potentially reflecting a lack for efficient self-regulation (Thayer et al. 2009; Koenig et al. 2017; Weise et al. 2020a, b). Longitudinal studies suggest that changes in adolescent resting-state HRV are associated with changes in BPD symptomatology (Koenig et al. 2018; Sigrist et al. 2021), and even predicted clinical symptom reduction in adolescent BPD patients receiving dialectical behavioural therapy (Weise et al. 2020b).

However, findings on resting-state HRV in BPD remain heterogeneous: Several studies failed to find a significant reduction of resting HRV in women with BPD as compared to healthy controls (Austin et al. 2007; Gratz et al. 2013; Meyer et al. 2016; Krause-Utz et al. 2019). Mixed findings can be attributed to the high heterogeneity of resting-state HRV among individuals with BPD (Koenig et al. 2020), as compared to healthy (Koenig et al. 2016) and other clinical conditions, such as post-traumatic stress disorder (Meyer et al. 2016). High heterogeneity in physiological baseline activity within groups of BPD and across studies seems to be a common phenomenon, equally observed when inspecting other biobehavioral indices linked to stress physiology (Scott et al. 2013; Wingenfeld et al. 2010; Ehrenthal et al. 2018). In a recent review, Koenig et al. (2020) concluded that these alterations in ANS function within individuals with BPD might be linked to severe experiences of early maltreatment and trauma.

### Heart-rate variability, childhood trauma and the moderating role of attachment insecurity

A history of *childhood traumatic experiences* and *attachment insecurity* are the most studied developmental risk factors of BPD (Gunderson et al. 2018; Ehrenthal et al. 2018), both being linked to disturbed vagal activity (Oosterman et al. 2010; Sigrist et al. 2020).

*Childhood trauma* is associated with a higher risk to develop BPD (see Gunderson et al. 2018 for review), with affected individuals being more than four times more likely to report childhood adversity as compared to non-clinical individuals (Porter et al. 2020; Kleindienst et al. 2020). Childhood trauma has been shown to exhaust early self-regulatory capacities and, therefore, contribute to the pathogenesis of dysregulated autonomic- and endocrine-functions in critical developmental periods, epigenetically priming physiological functioning towards heightened stress sensitivity (McLaughlin et al. 2015; Oosterman et al. 2010; Ferrer et al. 2021). Meyer et al. (2016) supported this strain of research concerning HRV as marker of ANS functioning, by showing that levels of childhood trauma were negatively associated with resting-state HRV in a sample including women with BPD. Childhood trauma might, therefore, generally prime the ANS towards higher sympathetic dominance, with different levels of early traumatization contributing to different HRV resting-states within BPD women (Meyer et al. 2016). Although childhood trauma can be considered a robust risk factor for BPD, early research already showed that some affected individuals nonetheless display a history of little or no traumatic experiences (Herman et al. 1989; Zanarini and Frankenburg 1997), thus childhood trauma does not necessarily and robustly precede BPD development (Gunderson et al. 2018). This suggests that additional mechanisms than only childhood trauma might be implicated in BPD pathogenesis.

A growing number of studies emphasizes that interindividual differences in *attachment insecurity* importantly contextualize the impact of childhood trauma on BPD symptoms (Crow and Levy 2019; Baryshnikov et al. 2017; Peng et al. 2021), such as on personality functioning (Gander et al. 2020) as well as on pathophysiological mechanisms of BPD related to sympathetic hyperactivity/stress sensitivity (Ehrenthal et al. 2018; Simeon et al. 2011). Children living in conditions with severe disruptions in the parent–child relationship (e.g., relational childhood trauma) are at greater risk to develop and maintain unresolved internal working models of social interactions, contributing to attachment insecurity and personality pathology until adulthood (Bowlby 1982; Liotti 2004; Cyr et al. 2010). Internal working models are conceptualized as cognitive self-regulatory mechanisms (Mikulincer et al. 2009). Therefore, individuals with very low levels of integration and coherence of internal working models (i.e. higher attachment insecurity) may have also developed an altered biological predisposition to stress regulation (Ehrenthal et al. 2018). Empirically, subgroups of severely insecure attached individuals with BPD show heightened cortisol activity related to heightened sympathetic activity in an interpersonal context (Ehrenthal et al. 2018). Concerning ANS functioning, insecurely attached individuals show reduced HRV when confronted with traumatic experiences, indicative for heightened sympathetic activity (Farina et al. 2015; Decarli et al. 2020). Although former psychophysiological studies support the moderating role of attachment insecurity when inspecting cortisol activity in women with BPD (Ehrenthal et al. 2018; Simeon et al. 2011), no study tested the moderating role of attachment security on the association between childhood trauma and resting-state HRV in individuals with BPD yet.

### Heart-rate variability, childhood trauma, attachment insecurity and oxytocin

A potential pharmacological intervention for reduced vagal activity as a physiological target mechanism underlying inefficient self-regulation in BPD might be intranasal administration of oxytocin (IN-OT; Herpertz and Bertsch 2015; Norman et al. 2011; Kemp et al. 2012). Oxytocin may especially impact the relationship between HRV, childhood trauma and attachment insecurity, as being conceptually and empirically linked to (1) the interaction between early-life stress and disturbed attachment in the pathogenesis of BPD (Herpertz and Bertsch 2015) and (2) IN-OT in dampening sympathetic activity at rest to facilitate capacity for adaptive self-regulatory behaviour (Norman et al. 2011; Kemp et al. 2012; Porges 2007).

Oxytocin is an important regulator of social cognition and behaviour (Kemp et al. 2012), implicated in successfully establishing early attachment relationship between child and caregivers following pregnancy (Rilling and Young 2014). According to a pathophysiological model of BPD (Herpertz and Bertsch 2015), BPD could both result from and result in low parental oxytocin levels, activating a neuroendocrine cascade associated with elevated risk for childhood stress, poor parental attachment, promoting attachment insecurity, hypersensitivity to stress, and impeding regulation capacities until adulthood. Indeed, reduced baseline oxytocin levels have been found in serum of women with BPD compared with HC (Bertsch et al. 2013a) and in cerebrospinal fluid of women with childhood trauma compared to women without childhood trauma (Heim et al. 2009).

IN-OT has been shown to reduce threat hypersensitivity and approach (Bertsch et al. 2013b; Schneider et al. 2019), such as amygdala and insular reactivity to stimuli of negative valence in women with BPD (Lischke et al. 2012). Oxytocin receptors are especially dense in specific limbic regions and their connections to the prefrontal cortex (Bartels and Zeki 2004), which may also modulate ANS activity (Fatisson et al. 2016). Peripherally, oxytocin receptors are located in the heart and surrounding vagal pathways. Thereby, oxytocin may upregulate parasympathetic and downregulate sympathetic responses, with IN-OT showing an enhancing effect on HRV at rest in healthy male individuals (Norman et al. 2011; Kemp et al. 2012). Although former studies support a beneficial effect of IN-OT on interpersonal pathology in BPD, no study investigated effects of IN-OT on intrapersonal mechanisms reflecting self-regulatory capacities, i.e. resting-state HRV in females with BPD, yet.

### The current study

Within the central project of the Clinical Research Unit 256 (Schmahl et al. 2014), the current study presents a secondary analysis of the subproject “Social Perception II”, encompassing a randomized-controlled trial studying the effects of oxytocin on social perception in BPD (Trial Registration Number: DRKS00009815). The aim of the present secondary investigation was to (1) replicate reduced baseline vagal activity in unmedicated BPD women as indexed by reduced time-domain measures of HRV (i.e. the root-mean-square of successive R–R-interval differences, RMSSD). RMSSD has been shown to be one of the most robust and reliable indicators of vagally mediated HRV and to be mostly unaffected by breathing artefacts (Penttilä et al. 2001). To further resolve the high level of heterogeneity in resting-state HRV activity within BPD women and across studies, we examined (2) the moderating role of adult attachment insecurity in the association between childhood trauma and RMSSD. However, we expect a smaller or no moderation effect within the sample of healthy women, in line with recent meta-analytic results showing a lack of relationship between CT and HRV in the absence of a concomitant psychopathological status (Sigrist et al. 2021). Lastly, we investigated (3) potential modulatory effects of oxytocin on RMSSD in women with BPD and healthy controls (HC) and (4) explored interactive effects of childhood trauma, attachment and clinical group with oxytocin on RMSSD.

## Materials

### Participants

A total of 53 medication-free adult female women with a current DSM-IV diagnosis of BPD (*M*_age_ = 30.02, SD = 7.42, range = 30) and 60 healthy female controls (*M*_age_ = 27.82, SD = 7.1, range = 34) participated in the study. Recruitment and diagnostic assessment were performed by the central project of the Clinical Research Unit 256 (Schmahl et al. 2014). The study was approved by the Ethics Committee of the Medical Faculty of the University of Heidelberg. All participants provided their written informed consent prior to study participation and were paid for their participation. All projects from this research group include participants from a joint database. Therefore, the BPD and HC sample was included in a publication of Schmitz et al. (2020) and Schneider et al. (2020) which, however, did not include investigation of HRV of patients with BPD compared to HC, which is the focus of the current study*.*

BPD women and healthy controls were matched for age (*t*(111) = 1.61, *p* = 0.109), and body mass index (BMI; calculated as body weight (kg)/body height (m)^2^) (*t*(111) = 1.5, *p* = 0.137) and did not differ in heart rate (mean = 69.4 bpm; *t*(111) = 1.8, *p* = 0.075). Both groups neither encountered any neurological disease, severe physical or psychiatric condition (including cardiac condition, pregnancy, current alcohol and/or drug abuse, drug and/or alcohol dependency 2 months prior testing, lifetime diagnosis of schizophrenia, schizoaffective or bipolar disorder), nor took any acute medication (except contraceptives and need-based medication, maximum 1 day prior to laboratory testing). Additionally, healthy controls did neither receive any lifetime psychiatric diagnosis, nor underwent any psychotherapeutic/psychiatric treatment.

### Materials

Qualified and trained diagnosticians assessed BPD and other psychiatric diagnoses with semi-structured interviews, i.e. the Structured Clinical Interview for DSM IV for Axis I disorders (SCID-VI-I; First et al. 1995) and the International Personality Disorder Examination for Axis II disorders (IPDE; Loranger et al. 1997).

Childhood traumatic experiences were assessed using the Childhood Trauma Questionnaire (CTQ; Bernstein et al. 2003). Participants rated the frequency of traumatic experiences on a five-point Likert scale (ranging from “never true” to “very often true”), for five scales (“physical abuse”, sexual abuse”, “emotional abuse”, “physical neglect” and “emotional neglect”). The total sum score was calculated, ranging from 25 to 125, with a Cronbach’s *α* of 0.87 in the present sample.

Adult romantic attachment style was operationalised by the Experiences in Close Relationships Scale (ECR-R; Ehrenthal et al. 2009), with the two subscales of anxiety and avoidance of attachment. A total mean score of the two scales was calculated, ranging from 1 to 7, indicative for the overall level of attachment insecurity, with a Cronbach’s *α* of 0.97 in the present sample.

A standardised questionnaire was used to measure demographic information, age, weight, height and BMI (see Table 1).

**Table 1:** Demographic, psychometric and self-reported data of women with borderline personality disorder (BPD) and healthy controls (HC) in the oxytocin (oBPD, *N* = 26; oHC, *N* = 30) and placebo condition (pBPD = 27; pHC, *N* = 30)

	oBPD (*n* = 26)	pBPD (*N* = 27)	oHC (*N* = 30)	pHC (*N* = 30)	BPD vs HC		oBPD vs. pBPD		oHC vs. pHC	
	*M* + SD	*M* + SD	*M* + SD	*M* + SD	*t*	*p*	*t*	*p*	*t*	*p*
Age (years)	29.0 + 7.9	31.0 + 6.9	27.6 + 7.8	28.0 + 6.4	1.62	0.109	− 1.02	0.313	− 0.20	0.842
Body mass index (BMI)	24.0 + 5.0	25.4 + 6.0	22.5 + 2.6	24.1 + 5.3	1.49	0.139	− 0.93	0.358	− 1.5	0.140
Heart rate (bpm)	69.0 + 8.4	73.0 + 9.6	65.2 + 9.2	70.3 + 10.5	1.80	0.075	− 1.58	0.121	− 2.03	0.048
BPD Dimensional Score (IPDE)	14.7 + 1.8	14.6 + 1.6	0.0 + 0.0	0.0 + 0.0	62.1	0.000	0.92	0.532	–	–
Early traumatization (CTQ)	58.9 + 22.2	63.0 + 18.1	31.2 + 6.3	31.4 + 9.3	9.96	0.000	− 0.73	0.467	− 0.10	0.921
Adult attachment style (ECR-R)	4.5 + 0.6	4.2 + 1.4	1.8 + 0.2	1.8 + 0.3	16.2	0.000	1.17	0.250	− 0.42	0.673

Explanatory note: Heart rates were calculated via ARTIIFACT (Kaufmann et al. 2011)

*BMI* body mass index, *BPD* women with borderline personality disorder, *CTQ* Childhood Trauma Questionnaire, *ECR-R* Experiences in Close Relationships Scale, *HC* healthy controls, *IPDE* International Personality Disorder Examination

### Procedure

After an extensive telephone screening, participants underwent a face-to-face clinical diagnostic interview. On the day of the experiment, participants were instructed not to smoke, take caffeine or analgesics and to relinquish eating 2 h prior to the experimental session. Testing took place in a sound-attenuated, dimly-lit room, followed by the collection of biological samples (not reported here). In a double-blind design, participants were randomly assigned via block randomisation (computerized algorithm, performed by an independent person not involved in the study), either to a single intranasal dose of 24 I.U. of oxytocin (six puffs of 2 I.U. to each nostril) or a placebo condition (spray with the same inactive ingredients but without oxytocin). The oxytocin and placebo nasal sprays were prepacked in identical bottles and consecutively numbered with each number being subdivided in “A” and “B” according to the randomisation schedule. The allocation sequence was concealed from both participant and the researcher conducting the study. Sealed and stapled envelopes, containing information about the allocation between number and content of the nasal spray, were locked and only opened after the last participant had completed the study. Electrodes were applied and participants performed several experimental tasks (see Schneider et al. 2020; Schmitz et al. 2020 for a detailed description). 85 min after spray administration, 4 min of ECG measurement were taken in a comfortable upright position, and participants were instructed to sit still and stay awake.

### Physiological data acquisition and processing

ECG was measured using 2 Ag/AgCl electrodes with micropore tape and solid gel (3 M Health Care) according to Eindhoven II configuration and stored at a sampling rate of 1000 Hz (72-chanel QuickAmp amplifier; Brain Products GmbH). Heart rate (HR) and R-peaks were detected offline with EDF-Browser Software (Version 1.71; van Beelen,^ 2019^) employing combined adaptive thresholding (Christov 2004). Raw ECG was visually inspected for artefacts and correct R-trigger location. Three participants (BPD = 1, HC = 2) had to be excluded from further HRV-Analysis due to poor raw ECG signal quality and concomitant fail of automatic R-Trigger detection. Inter-beat-intervals (IBI) were transferred to ASCII format, cleaned and processed using the RHRV package, an automatic tool for HRV analysis in R (Martínez et al. 2017). Cleaned and processed IBIs were additionally visually reviewed for potential artefacts. In a final step, RMSSD indices were derived automatically and transferred to SPSS (IBM SPSS 26). Zhang and colleagues suggested that RHRV-extracted indices for HRV display high reliability and validity (*r* > 0.8) compared to respective indices retrieved from the gold-standard software Kubios (Zhang et al. 2020).

### Statistical analysis

Group differences in RMSSD (*hypothesis 1*) as well as effects of oxytocin administration on RMSSD (*hypothesis 3*) were analysed using a 2 × 2 analysis of variance (ANOVA; SPSS 26), with the between-subject factors group (BPD, HC) and substance (oxytocin, placebo). A moderated linear regression analysis using the PROCESS plug-in for SPSS was performed (model number 1, version 3.4.1; Hayes 2017) to test for a potential moderating effect of attachment insecurity score on the association between the childhood trauma score and mean RMSSD score in the BPD group, and in the HC group (all predictors were mean centred; *hypothesis 2*). Moreover, we exploratory tested for potential interactions of childhood trauma, attachment insecurity, substance and clinical status on RMSSD (exploratory question *4*) by running an ANCOVA with the between-subjects factor group (BPD, HC), substance (oxytocin, placebo) as well as childhood trauma (continuously) and attachment (continuously) as covariates, checking for every specific interaction with substance (childhood trauma*substance, attachment*substance, childhood trauma*attachment*substance, childhood trauma*attachment*group*substance). Partial eta-squared (*η*^2^) are reported as effect size (0.01, 0.06, 0.14 are considered small, medium and large effects, respectively (Cohen 1988)).

## Results

**Fig. 1:**
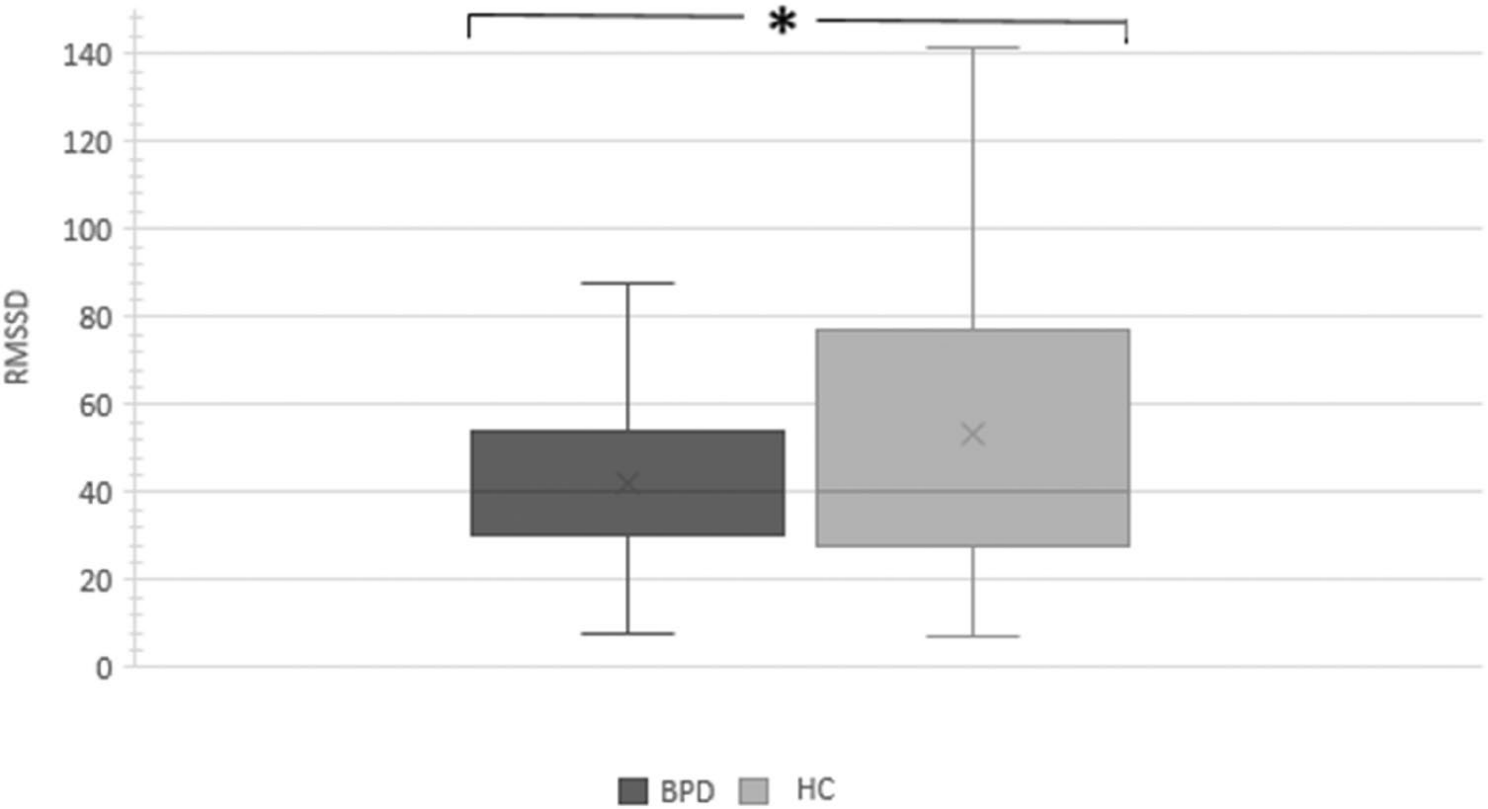
Box-plot diagram of the RMSSD of women with current borderline personality disorder (BPD; *N* = 52), and healthy controls (HC; *N* = 58). *p > 0.05

As expected (*hypothesis 1*), women with BPD had significantly lower mean RMSSD values than HC (*F*(1,107) = 4.6; *p* = 0.034, *η*^2^ = 0.04, see Fig. 1). Concerning *hypothesis 2*, neither the main effect of substance (*F*(1,107) = 2.4, *p* = 0.121, *η*^2^ = 0.02) nor the group by substance interaction (*F*(1,107) = 0.5; *p* = 0.475, *η*^2^ = 0.01) reached statistical significance. Thus, contrary to *hypothesis 3*, there was no evidence that IN-OT modulated HRV activity in the current study, neither in women with BPD, nor HC.

**Fig. 2:**
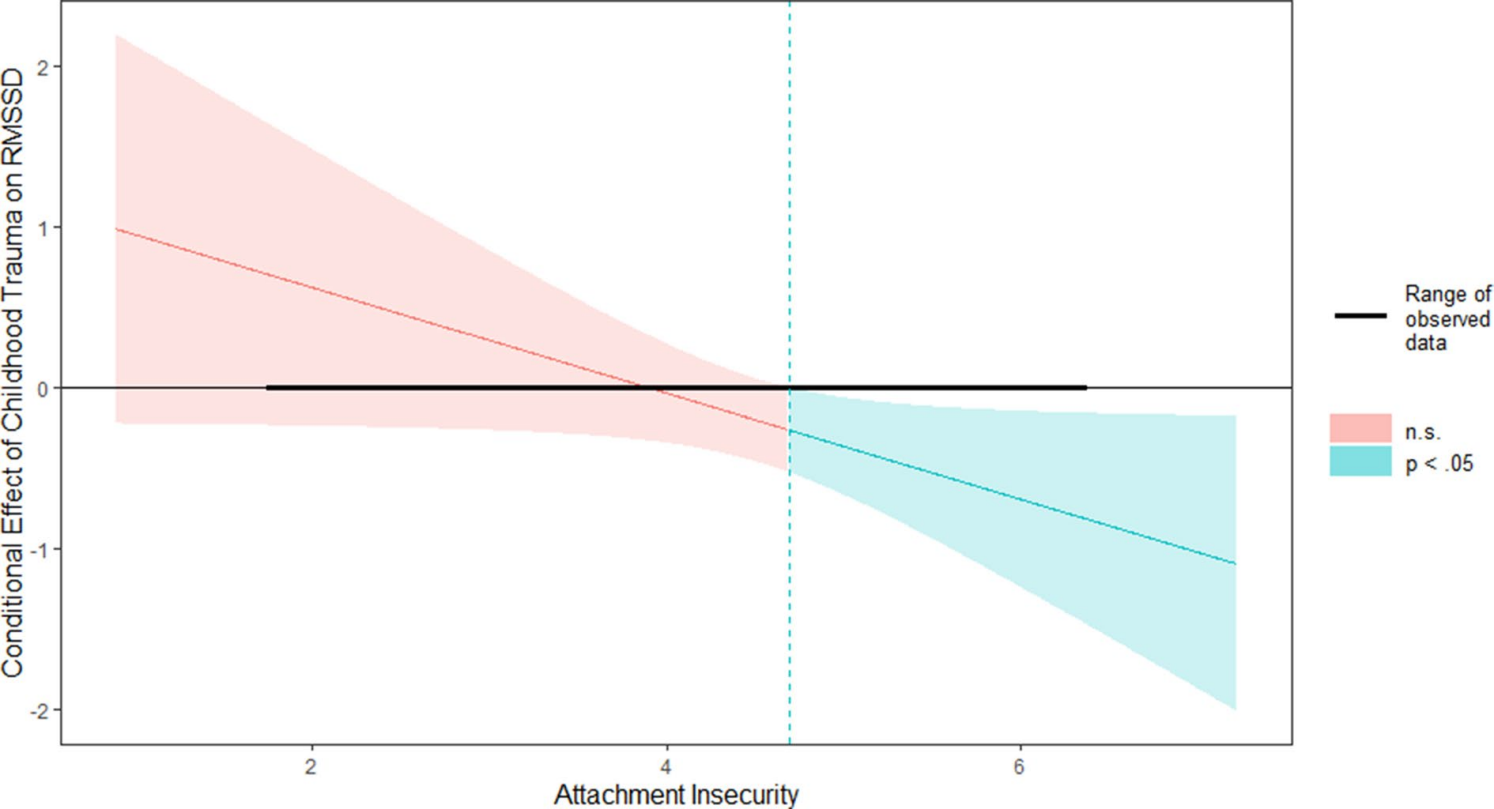
Johnson–Neyman plot of the conditional effect of *X* (= Childhood Trauma, CTQ) on *Y* (= HRV, RMSSD) of moderator (= Attachment Insecurity, ECR). The dashed blue vertical line (mean attachment score = 4.69) represents the point where the relationship between Childhood Trauma and RMSSD transitions from statistically non-significant (red) to significant (blue) according to Johnson–Neyman analysis. Note: The range of observed values of attachment insecurity (ECR) is [1.73; 6.36]

Concerning *hypothesis 2*, moderated regression analysis for the BPD group revealed that the *R*^2^ for the overall model within the BPD group was *R*^2^ = 0.16 (adjusted *R*^2^ = 0.11), indicative for a medium goodness-of-fit (*R*^2^ = 0.16, *F*(3,48) = 3.15, *p* = 0.033) according to Cohen (1988). Hence, the model including the three variables “childhood trauma”, “attachment” and “childhood trauma*attachment” explained 16% of the total variance in RMSSD in BPD women. At the individual level of the predictors, “attachment” (*R*^2^ = 0.16, *F*(3,48) = 3.15, *p* = 0.033, *b* = 0.18, *t*(48) = 2.08, *p* = 0.042) and the interaction “childhood trauma*attachment” (*R*^2^ = 0.16, *F*(3,48) = 3.15, *p* = 0.03, *b* = − 0.01, *t*(48) = 2.03, *p* = 0.048) significantly predicted RMSSD in the BPD group. Concerning the predictor “attachment”, the regression indicated that higher levels of attachment insecurity were linearly associated with lower basal HRV levels in women with BPD. The interaction term between childhood trauma and attachment accounted significantly for 7% of the total variance in RMSSD (Δ*R*^2^ = 0.07, Δ*F*(3,48) = 3.15, *p* = 0.03, *b* = − 0.01, *t*(48) = 2.03, *p* = 0.048). Johnson–Neyman technique revealed a significant interaction between childhood trauma and RMSSD only when attachment values exceeded a total mean score of 4.69 (*p* = 0.05; 40.38% of BPD sample, see Fig. 2). When the mean attachment score is below the respective interval [4.69; 6.36], the slope of childhood trauma on RMSSD was non-significant (*p* = 0.110–0.900). Therefore, the moderation analysis revealed that the association between childhood trauma and HRV levels was significant in those women with BPD demonstrating high levels of attachment insecurity. Thus, in accordance with *hypothesis 2*, attachment insecurity moderated the relationship between childhood trauma and HRV in women with BPD.

The respective moderated regression model for the HC group did not reach statistical significance (*R*^2^ = 0.09, *F*(1,55) = 7.2, *p* = 0.540). As expected, (see *hypothesis 2*), the predictive model between CT, attachment insecurity and HRV does not seem to apply for healthy women, due to a lack of variance within the predictors.

With regard to *exploratory hypothesis 4*, we found no interaction including the factor substance: neither the interaction substance by childhood trauma (*F*(2,108) = 1.019; *p* = 0.365), substance by attachment (*F*(2,108) = 0.121; *p* = 0.886), substance by childhood trauma by attachment (*F*(2,108) = 0.538; *p* = 0.585), or substance by childhood trauma by attachment by group (*F*(2,108) = 0.345; *p* = 0.709). Thus, there was no evidence that IN-OT modulated HRV depending on different levels of attachment insecurity, childhood trauma, or having a diagnosis of BPD or not.

## Discussion

The aim of the current study was to (1) replicate findings on reduced HRV at rest in women with BPD compared to HC, and to test if the (2) interaction between childhood trauma and attachment insecurity predicted reduced HRV in women with BPD and HC. Moreover, we investigated (3) modulations of oxytocin administration on HRV at rest and (4) explored interactions with childhood trauma, attachment insecurity and clinical status.

Most importantly, a significantly diminished HRV at rest in women with BPD compared to healthy women was predicted by interactions between high attachment insecurity and increased childhood trauma in the current sample. Only individuals with BPD and high attachment insecurity showed a significantly negative association between childhood trauma and HRV at rest. We found no evidence for linear relationships between CT and attachment insecurity predicting HRV, neither when accounting for a potential modulation between those predictors, in healthy women. Moreover, (3) IN-OT had no effect on HRV at rest, neither in the BPD, nor in the HC group and this lack of effect remained stable after (4) exploring interactions with childhood trauma and attachment insecurity.

In line with *hypothesis 1*, women with BPD showed significantly reduced vagally mediated HRV as indexed by a commonly reported time domain measures RMSSD, compared to HC. Our result of reduced resting HRV in BPD women is in line with the results of Koenig et al. (2016), but the effects of the current study were smaller than the medium-sized effects as proposed by meta-analysis (Koenig et al. 2016). Reduced baseline autonomic functioning in BPD could display a chronic state of physiological imbalance towards less vagal activity, reflecting reduced biological capacity for efficient self-regulatory mechanisms (Thayer and Brosschot 2005; Austin et al. 2007). Whereas we found a small but significant difference in HRV, many other studies reported no significant differences in HRV between BPD and HC (Meyer et al. 2016; Austin et al. 2007; Gratz et al. 2013). The differences in results and effect sizes might be explained by the inconsistency in study design, assessed confounding factors (e.g., physical activity), BPD sample characteristics (e.g., medication) and size, as well as indices of HRV throughout the studies, calling for more replicability in HRV and BPD research designs.

The current results provide evidence for childhood trauma and attachment insecurity as relevant predictors explaining 16% of HRV variance in women with BPD. In line with *hypothesis 2*, we found that higher reports of childhood trauma predicted lower basal HRV in BPD, but only in those individuals with high levels of acute attachment insecurity, and no linear relationship between those constructs within the HC sample.

In the light of the neurovisceral integration model (Thayer and Brosschot 2005), a chronic state of sympathetic stress mobilization may represent a maladaptive physiological threat adaptation resulting from a combination of very early traumatic experiences and the presence of insecurity in current relationships. These interpretations are in line with previous research suggesting an interactive influence of childhood trauma and adult attachment insecurity on BPD symptoms (Carlson et al. 2009; Baryshnikov et al. 2017; Godbout et al. 2019), as well as the psychophysiological results pointing to the specific role of unresolved attachment on heightened stress sensitivity in BPD (Simeon et al. 2011). The lack of significant regressions within the HC group provides further evidence for the above-mentioned moderation to interact with psychopathological status. This is in line with the results of a current meta-analysis (Sigrist et al. 2020) which outlined the evidence of the negative relationship between childhood trauma and HRV only in the presence of psychopathology. Due to the almost absence of traumatic childhood experiences and attachment insecurity in the healthy control group (see Table 1), the variance in those predictors might have most likely been too low to explain sufficient variance within HRV for the regression model to become significant.

In conclusion, our results provide further support for the relevance of developmental constructs in contextualizing biobehavioural mechanisms and interindividual variations in BPD (Ehrenthal et al. 2018). Following the interpretations of Sigrist et al. (2020), we suppose that CT might potentially contribute to alterations within ANS development in childhood, as indexed by low vagal activity (low HRV), eventually perpetuating into adulthood as a function of acute insecurity in current relationships, and increasing the risk for psychopathology related to self- and interpersonal dysfunctioning (e.g., BPD).

Contrary to *hypothesis 3*, resting-state HRV was not modulated by oxytocin administration, as there was neither a significant main effect of substance, nor an interaction effect with clinical status. In contrast to our results, intranasal administration of oxytocin has been found to enhance baseline HRV in male HC (Norman et al. 2011; Kemp et al. 2012) as well as in a male clinical sample (Martins et al. 2020). As recent studies point to a pattern of interactive effects with childhood trauma and attachment insecurity (Schoormans et al. 2020; Riem et al. 2021), we conducted exploratory analysis (*hypothesis 4*) but found no significant interaction of neither childhood trauma, attachment insecurity nor clinical group with oxytocin on RMSSD at rest. In contrast, it has been suggested that oxytocin is involved in attachment security and that methylation of the oxytocin receptor may be involved in the epigenetic modulation of childhood trauma (see Brüne 2016 for a review).

The lack of IN-OT effect is generally obedient with the *social salience hypothesis*, which states that oxytocin solely impacts neurophysiological activity under circumstances of salient social cues (Wigton et al. 2015). Our findings match those reported by Schmitz et al. (2020), who did not find alterations in heartbeat evoked brain potentials at rest following oxytocin administration in BPD women within the same sample. Moreover, our results match the interpretations by Riem et al. (2021), attributing altered HRV after oxytocin administration to an increased attentional salience as induced by the social presence during the experiment. Concerning the interactive effects with early trauma, in accordance to our results, childhood trauma did not modulate IN-OT on salivary oxytocin in male adolescents (Fragkaki et al. 2020) and HRV was unaffected by IN-OT in individuals with adverse childhood experiences (Schoormans et al. 2020). Matching the conclusions as initially drawn by Herpertz and Bertsch (2015), IN-OT might, therefore, be more suited in improving BPD associated symptoms/mechanisms (including disturbed vagal activity) in the context of *social interactions* with and attachment to familiar people (e.g., before seeing a psychotherapist). Study designs following this hypothesis would be of interest.

Several limitations should be considered. First, we did not control for physical activity, smoking and menstrual cycle, as well as age and BMI, despite official recommendations for their inclusion into HRV research (Quintana et al. 2016). Therefore, we cannot exclude the possibility that differences in HRV between groups and their interaction with oxytocin and developmental constructs might be influenced by the above-mentioned intervening variables. Nevertheless, the groups were matched according to age, gender, and did not differ in terms of heart rate or BMI. Moreover, we only investigated RMSSD as indicator of HRV activity, although frequency domain indicators (such as high-frequency HRV) are also adequate, especially in capturing vagal activity (Task Force of the European Society of Cardiology 1996). As we only measured ECG for 4 min, and did not control for breathing artifacts, we decided to rely on a relatively robust, single indicator of HRV (e.g., RMSSD, Penttilä et al. 2001; Quintana et al. 2016). The interpretations concerning the lacked oxytocin effect should be treated with caution: Most studies which found an effect of IN-OT on resting HRV relied on male individuals (Norman et al. 2011; Kemp et al. 2012), and those studies on females controlled for menstrual cycle (Schoormans et al. 2020), which we did not. Therefore, possible effects of oxytocin on HRV in females with BPD might eventually be present, as a function of menstrual cycle. Last, we employed a cross-sectional design, including the well documented limitations for reporting past traumatization (Maughan and Rutter 1997), hindering causal inference.

We conclude that reduced vagal activity (e.g., reduced HRV) in BPD might be a promising target of intervention, as being associated with severity of early traumatic experiences in insecurely attached females with BPD. Future studies should aim to replicate our findings controlling for important covariates influencing HRV (e.g., physical activity, smoking, breath) employing longitudinal research designs. Moreover, our results suggest that IN-OT may not be a suitable intervention in enhancing autonomic functioning at rest in women with BPD. Further research on female individuals with BPD, controlling for menstrual cycle and employing interpersonal contexts are needed to draw clear conclusions on oxytocin’s pharmacological potential.

## Data Availability

The datasets generated during and/or analysed during the current study are available from the corresponding author on reasonable request.

## References

[CR1] Austin MA, Riniolo TC, Porges SW (2007). Borderline personality disorder and emotion regulation: Insights from the Polyvagal Theory. Brain Cogn.

[CR2] Bartels A, Zeki S (2004). The neural correlates of maternal and romantic love. Neuroimage.

[CR3] Baryshnikov I, Joffe G, Koivisto M, Melartin T, Aaltonen K, Suominen K, Isometsä E (2017). Relationships between self-reported childhood traumatic experiences, attachment style, neuroticism and features of borderline personality disorders in patients with mood disorders. J Affect.

[CR4] Beauchaine TP, Thayer JF (2015). Heart rate variability as a transdiagnostic biomarker of psychopathology. Int J Psychophysiol.

[CR5] Berntson GG, Thomas Bigger J, Eckberg DL, Grossman P, Kaufmann PG, Malik M, Nagaraya HN, Porges SW, Saul JP, Stone PH, van der Molen MW (1997). Heart rate variability: origins, methods, and interpretive caveats. J Psychophysiol.

[CR6] Bertsch K, Schmidinger I, Neumann ID, Herpertz SC (2013). Reduced plasma oxytocin levels in female patients with borderline personality disorder. Horm Behav.

[CR7] Bertsch K, Gamer M, Schmidt B, Schmidinger I, Walther S, Kästel T, Herpertz SC (2013). Oxytocin and reduction of social threat hypersensitivity in women with borderline personality disorder. Am J Psychiatry.

[CR8] Bowlby J (1982). Attachment and loss: retrospect and prospect. Am J Orthopsychiatry.

[CR9] Brüne M (2016). On the role of oxytocin in borderline personality disorder. Br J Clin Psychol.

[CR10] Carlson EA, Egeland B, Sroufe LA (2009). A prospective investigation of the development of borderline personality symptoms. Dev Psychopathol.

[CR11] Christov II (2004). Real time electrocardiogram QRS detection using combined adaptive threshold. Biomed Eng Online.

[CR12] Cohen J (1988). The effect size. Statistical power analysis for the behavioral sciences.

[CR13] Crow TM, Levy KN (2019). Adult attachment anxiety moderates the relation between self-reported childhood maltreatment and borderline personality disorder features. Personal Ment Health.

[CR14] Cyr C, Euser EM, Bakermans-Kranenburg MJ, Van Ijzendoorn MH (2010). Attachment security and disorganization in maltreating and high-risk families: a series of meta-analyses. Dev Psychopathol.

[CR15] Decarli A, Pierrehumbert B, Schulz A, Schaan VK, Vögele C (2020). Disorganized attachment in adolescence: emotional and physiological dysregulation during the Friends and Family Interview and a conflict interaction. Dev Psychopathol.

[CR16] Ehrenthal JC, Dinger U, Lamla A, Funken B, Schauenburg H (2009). Evaluation der deutschsprachigen Version des Bindungsfragebogens “Experiences in Close Relationships–Revised” (ECR-RD). PPmP-Psychotherapie·psychosomatik Medizinische Psychologie.

[CR17] Ehrenthal JC, Levy KN, Scott LN, Granger DA (2018). Attachment-related regulatory processes moderate the impact of adverse childhood experiences on stress reaction in borderline personality disorder. J Personal Dirsord.

[CR18] Farina B, Speranza AM, Imperatori C, Quintiliani MI, Marca GD (2015). Change in heart rate variability after the Adult Attachment Interview in dissociative patients. J Trauma Dissociation.

[CR19] Fatisson J, Oswald V, Lalonde F (2016). Influence diagram of physiological and environmental factors affecting heart rate variability: an extended literature overview. Heart Int.

[CR20] Ferrer A, Soria V, Salvat-Pujol N, Martorell L, Armario A, Urretavizcaya M, Labad J (2021). The role of childhood trauma, HPA axis reactivity and FKBP5 genotype on cognition in healthy individuals. Psychoneuroendocrinology.

[CR21] First MB, Spitzer RL, Gibbon M, Williams JB (1995). The structured clinical interview for DSM-III-R personality disorders (SCID-II). Part I: description. J Personal Disord.

[CR23] Fragkaki I, Glennon JC, Cima M (2020). Salivary oxytocin after oxytocin administration: examining the moderating role of childhood trauma. Biol Psychol.

[CR24] Gander M, Buchheim A, Bock A, Steppan M, Sevecke K, Goth K (2020). Unresolved attachment mediates the relationship between childhood trauma and impaired personality functioning in adolescence. J Personal Disord.

[CR25] Godbout N, Daspe M-È, Runtz M, Cyr G, Briere J (2019). Childhood maltreatment, attachment, and borderline personality-related symptoms: gender-specific structural equation models. Psychol Trauma.

[CR26] Gratz KL, Tull MT, Matusiewicz AM, Breetz AA, Lejuez CW (2013). Multimodal examination of emotion regulation difficulties as a function of co-occurring avoidant personality disorder among women with borderline personality disorder. Personal Disord.

[CR27] Gunderson JG, Herpertz SC, Skodol AE, Torgersen S, Zanarini MC (2018). Borderline personality disorder. Nat Rev Dis Prim.

[CR28] Hayes AF (2017). Introduction to mediation, moderation, and conditional process analysis: a regression-based approach.

[CR29] Heim C, Young LJ, Newport DJ, Mletzko T, Miller AH, Nemeroff CB (2009). Lower CSF oxytocin concentrations in women with a history of childhood abuse. Mol Psychiatry.

[CR30] Herman JL, Perry JC, Van der Kolk BA (1989). Childhood trauma in borderline personality disorder. Am J Psychiatry.

[CR31] Herpertz SC, Bertsch K (2015). A new perspective on the pathophysiology of borderline personality disorder: a model of the role of oxytocin. Am J Psychiatry.

[CR32] Kaufmann Tobias, Sütterlin Stefan, Schulz Stefan M., Vögele Claus (2011). ARTiiFACT: a tool for heart rate artifact processing and heart rate variability analysis. Behavior Research Methods.

[CR33] Kemp AH, Quintana DS, Kuhnert RL, Griffiths K, Hickie IB, Guastella AJ (2012). Oxytocin increases heart rate variability in humans at rest: implications for social approach-related motivation and capacity for social engagement. PLoS ONE.

[CR34] Kleindienst N, Vonderlin R, Bohus M, Lis S (2020). Childhood adversity and borderline personality disorder. Analyses complementing the meta-analysis by Porter et al., 2020. Acta Psychiatr Scand.

[CR35] Koenig J, Kemp AH, Feeling NR, Thayer JF, Kaess M (2016). Resting state vagal tone in borderline personality disorder: a meta-analysis. Prog Neuropsychopharmacol Biol Psychiatry.

[CR36] Koenig J, Rinnewitz L, Parzer P, Resch F, Thayer JF, Kaess M (2017). Resting cardiac function in adolescent non-suicidal self-injury: the impact of borderline personality disorder symptoms and psychosocial functioning. Psychiatry Res.

[CR37] Koenig J, Weise S, Rinnewitz L, Parzer P, Resch F, Kaess M (2018). Longitudinal covariance of resting-state cardiac function and borderline personality disorder symptoms in adolescent non-suicidal self-injury. World J Biol Psychiatry.

[CR38] Koenig J, Thayer JF, Kaess M (2020). Psychophysiological concomitants of personality pathology in development. Curr Opin Psychol.

[CR39] Krause-Utz A, Walther J, Lis S, Schmahl C, Bohus M (2019). Heart rate variability during a cognitive reappraisal task in female patients with borderline personality disorder: the role of comorbid posttraumatic stress disorder and dissociation. Psychol Med.

[CR40] Lane RD, McRae K, Reiman EM, Chen K, Ahern GL, Thayer JF (2009). Neural correlates of heart rate variability during emotion. Neuroimage.

[CR41] Liotti G (2004). Trauma, dissociation, and disorganized attachment: three strands of a single braid. Psychol Psychother.

[CR42] Lischke A, Gamer M, Berger C, Grossmann A, Hauenstein K, Heinrichs M, Domes G (2012). Oxytocin increases amygdala reactivity to threatening scenes in females. Psychoneuroendocrinology.

[CR43] Loranger AW, Janca A, Sartorius N (1997). Assessment and diagnosis of personality disorders: the ICD-10 international personality disorder examination (IPDE).

[CR44] Martínez CAG, Quintana AO, Vila XA, Touriño MJL, Rodríguez-Liñares L, Presedo JMR, Penín AJM (2017). Heart rate variability analysis with the R package RHRV.

[CR45] Maughan B, Rutter M (1997). Retrospective reporting of childhood adversity: issues in assessing long-term recall. J Personal Disord.

[CR46] McLaughlin KA, Sheridan MA, Tibu F, Fox NA, Zeanah CH, Nelson CA (2015). Causal effects of the early caregiving environment on development of stress response systems in children. Proc Natl Acad Sci India A.

[CR47] Meyer PW, Müller LE, Zastrow A, Schmidinger I, Bohus M, Herpertz SC, Bertsch K (2016). Heart rate variability in patients with post-traumatic stress disorder or borderline personality disorder: relationship to early life maltreatment. J Neural Transm.

[CR48] Mikulincer M, Shaver PR, Cassidy J, Berant E, Obegi JH, Berant E (2009). Attachment-related defensive processes. Attachment theory and research in clinical work with adults.

[CR49] Norman GJ, Cacioppo JT, Morris JS, Malarkey WB, Berntson GG, DeVries AC (2011). Oxytocin increases autonomic cardiac control: moderation by loneliness. Biol Psychol.

[CR50] Oosterman M, De Schipper JC, Fisher P, Dozier M, Schuengel C (2010). Autonomic reactivity in relation to attachment and early adversity among foster children. Dev Psychopathol.

[CR51] Ottaviani C, Zingaretti P, Petta AM, Antonucci G, Thayer JF, Spitoni GF (2018). Resting heart rate variability predicts inhibitory control above and beyond impulsivity. J Psychophysiol.

[CR52] Peng W, Liu Z, Liu Q, Chu J, Zheng K, Wang J, Yi J (2021). Insecure attachment and maladaptive emotion regulation mediating the relationship between childhood trauma and borderline personality features. Depress Anxiety.

[CR53] Penttilä J, Helminen A, Jartti T, Kuusela T, Huikuri HV, Tulppo MP, Scheinin H (2001). Time domain, geometrical and frequency domain analysis of cardiac vagal outflow: effects of various respiratory patterns. Clin Physiol.

[CR54] Porges SW (2007). The Polyvagal perspective. Biol Psychol.

[CR55] Porter C, Palmier-Claus J, Branitsky A, Mansell W, Warwick H, Varese F (2020). Childhood adversity and borderline personality disorder: a meta-analysis. Acta Psychiatr Scand.

[CR56] Quintana DS, Alvares GA, Heathers JAJ (2016). Guidelines for Reporting Articles on Psychiatry and Heart rate variability (GRAPH): recommendations to advance research communication. Transl Psychiatry.

[CR57] Riem MME, Kunst LE, Kop WJ (2021). Intranasal oxytocin and the stress-buffering effects of social support during experimentally induced pain: the role of attachment security. J Affect.

[CR58] Rilling JK, Young LJ (2014). The biology of mammalian parenting and its effect on offspring social development. Science.

[CR59] Schmitz M, Müller LE, Schulz A, Kleindienst N, Herpertz SC, Bertsch K (2020). Heart and brain: cortical representation of cardiac signals is disturbed in borderline personality disorder, but unaffected by oxytocin administration. J Affect.

[CR60] Schneider I, Boll S, Volman I, Roelofs K, Spohn A, Herpertz SC, Bertsch K (2020). Oxytocin normalizes approach—avoidance behavior in women with borderline personality disorder. Front Psychiatry.

[CR61] Schoormans D, Kop WJ, Kunst LE, Riem MME (2020). Oxytocin effects on resting-state heart rate variability in women: the role of childhood rearing experiences. Compr Psychoneuroendocrinol.

[CR62] Scott LN, Levy KN, Granger DA (2013). Biobehavioral reactivity to social evaluative stress in women with borderline personality disorder. Personal Disord Theory Res Treat.

[CR63] Sigrist C, Mürner-Lavanchy I, Peschel SK, Schmidt SJ, Kaess M, Koenig J (2020). Early life maltreatment and resting-state heart rate variability: a systematic review and meta-analysis. Neurosci Biobehav Rev.

[CR64] Sigrist C, Reichl C, Schmidt SJ, Brunner R, Kaess M, Koenig J (2021). Cardiac autonomic functioning and clinical outcome in adolescent borderline personality disorder over two years. Prog Neuropsychopharmacol Biol Psychiatry.

[CR65] Simeon D, Bartz J, Hamilton H, Crystal S, Braun A, Ketay S, Hollander E (2011). Oxytocin administration attenuates stress reactivity in borderline personality disorder: a pilot study. Psychoneuroendocrinology.

[CR22] Task Force of the European Society of Cardiology and the North American Society of Pacing and Electrophysiology (1996) Heart rate variability: Standards of measurement, physiological interpretation, and clinical use. Eur Heart J 17:354–381 8737210

[CR66] Thayer JF, Brosschot JF (2005). Psychosomatics and psychopathology: looking up and down from the brain. Psychoneuroendocrinology.

[CR67] Thayer JF, Hansen AL, Saus-Rose E, Johnsen BH (2009). Heart rate variability, prefrontal neural function, and cognitive performance: the neurovisceral integration perspective on self-regulation, adaptation, and health. Ann Behav Med.

[CR68] Van Beelen, T (2019) EDFBrowser. Available online at: https://www.teuniz.net/edfbrowser/ (Accessed December, 2019)

[CR69] Weise S, Parzer P, Fürer L, Zimmermann R, Schmeck K, Resch F, Koenig J (2020). Autonomic nervous system activity and dialectical behavioral therapy outcome in adolescent borderline personality pathology. World J Biol Psychiatry.

[CR70] Weise S, Parzer P, Zimmermann R, Fürer L, Resch F, Kaess M, Koenig J (2020). Emotion dysregulation and resting-state autonomic function in adolescent borderline personality disorder—a multimodal assessment approach. Personal Disord Theory Res Treat.

[CR71] Wigton R, Radua J, Allen P, Averbeck B, Meyer-Lindenberg A, McGuire P, Shergill SS, Fusar-Poli P (2015). Neurophysiological effects of acute oxytocin administration: systematic review and meta-analysis of placebo-controlled imaging studies. J Psychiatry Neurosci.

[CR72] Wingenfeld K, Spitzer C, Rullkötter N, Löwe B (2010). Borderline personality disorder: hypothalamus pituitary adrenal axis and findings from neuroimaging studies. Psychoneuroendocrinology.

[CR73] Zanarini MC, Frankenburg FR (1997). Pathways to the development of borderline personality disorder. J Personal Disord.

[CR74] Zhang N, Hoch J, Gewirtz AH (2020). The physiological regulation of emotion during social interactions: vagal flexibility moderates the effects of a military parenting intervention on father involvement in a randomized trial. Prev Sci.

